# Porosity at Different Structural Levels in Human and Yak Belly Hair and Its Effect on Hair Dyeing

**DOI:** 10.3390/molecules25092143

**Published:** 2020-05-03

**Authors:** Alexander R. M. Müllner, Ruben Pahl, Doris Brandhuber, Herwig Peterlik

**Affiliations:** 1 Faculty of Physics, University of Vienna, Boltzmanngasse 5, A-1090 Vienna, Austria; alexander.muellner@univie.ac.at; 2 LIM Cosmetics GmbH, Fugbachgasse 17/3-5, A-1020 Vienna, Austria; ruben@lessismore.at (R.P.); doris@lessismore.at (D.B.)

**Keywords:** hair, nanostructure, mechanical properties, SAXS, hair dye, porosity

## Abstract

Yak belly hair was proposed as a cheap substitute for human hair for the development of hair dyes, as its chemical composition closely resembles human hair in Raman spectroscopy. The absence of melanin in yak belly hair also leads to a strong reduction of fluorescence in Raman measurements, which is advantageous for the investigation of the effectivity of hair dyes. To assess the suitability for replacing human hair, we analyzed similarities and differences of both hair types with a variety of methods: Raman spectroscopy, to obtain molecular information; small-angle X-ray scattering to determine the nanostructure, such as intermediate filament distance, distance of lipid layers and nanoporosity; optical and scanning electron microscopy of surfaces and cross sections to determine the porosity at the microstructural level; and density measurements and tensile tests to determine the macroscopic structure, macroporosity and mechanical properties. Both types of hair are similar on a molecular scale, but differ on other length scales: yak belly hair has a smaller intermediate filament distance on the nanoscale. Most striking is a higher porosity of yak belly hair on all hierarchical levels, and a lower Young’s modulus on the macroscale. In addition to the higher porosity, yak belly hair has fewer overlapping scales of keratin, which further eases the uptake of coloring. This makes, on the other hand, a comparison of coloring processes difficult, and limits the usefulness of yak belly hair as a substitute for human hair.

Academic Editors: Helga Lichtenegger and Harald Rennhofer

## 1. Introduction

Hair is a proteinaceous fiber of mammals, which serves a wide range of purposes: it acts as mechanoreceptor in the form of whiskers [1]; as thermal insulation material, e.g., wool and polar bear hair [2]; and/or water protection (in particular for water living species such as beavers or water shrews [3]); or as particle filters, e.g., eyelashes, nose and ear hair [4]. For human beings, it is furthermore a widely used symbol of strength, power and beauty [5], and can easily be varied, which offers the opportunity of coloring, styling and designing the appearance. The interest in different hair colors has a long tradition—it is already documented in ancient Rome, though at that time wigs were used [6]. It is only natural that the cosmetic industry has a high interest in the development of new products for different approaches to shape and style hair. These products require a large number of hair strands for development and for testing the suitability and effectivity of the products. Additionally, for quality control, hair strands with constant sample properties would be an advantage.

One of the most widely used applications is dyes for hair coloring. Coloring can be performed either on natural or on bleached hair. The latter opens a much wider color spectrum, though it could damage the hair structure. Pudney et al. [7] used Raman spectroscopy of whole hairs to investigate the effect of bleaching. They found that this procedure mainly affects the outer cuticle layer [7], and they also proposed that yak hair could be a good and cheap alternative to human hair, due to its lack in melanin [7]. This was the starting point for our paper: to assess the quality of yak hair to substitute human hair, we characterized yak belly hair and human hair with a number of different methods. As hair is a complex structure with a hierarchical organization of subunits from the α-keratin chains via intermediate filaments to the fiber [5], the characterization methods should also cover this broad range from the nano- via the meso- to the macroscale. Chemically, hair is for the most part (65%–95%) built from keratin [8] and has three different regions, from the center to the outside medulla, cortex and cuticle. The cuticle of human hair consists of flat cells that are arranged like tiles on a roof or fish scales and serve a protective purpose [5].

**Figure 1:**
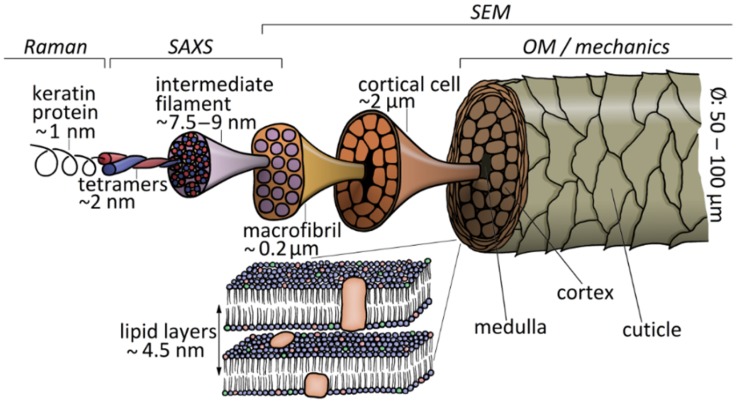
Hierarchical structure of hair under various magnifications. The size range accessible to the respective test method is also indicated in the figure.

Figure 1 shows a sketch of the structure of hair under various magnifications. Molecular information on the single keratin fibers, such as chemical bonds and conformation [9], as well as internal structural changes [10], is obtained from Raman spectroscopy. The basic structural feature at the nanoscale are the keratin intermediate filaments (IF), whose structure and framework were described in a pioneering early work [11]. An overview on the architecture and assembly of IF is found in [12], and the molecular packing of IF and possible positions of crosslinks between IF by disulfide bonds were modelled by Fraser et al. [13,14,15]. Transmission electron microscopy is now able to visualize the double-twist three-dimensional architecture of macrofibril-bundles of IF [16]. The typical IF to IF distance is most easily evaluated from small-angle X-ray scattering (SAXS) data [17]. In [17], also wide-angle X-ray scattering (WAXS) was used for the analysis of the submolecular structure of variations in different ethnic hair types. SAXS gives information on the distance of lipid bilayers, which were assumed to be present in the cell membrane complex. The bilayers have a typical distance of 4.5 nm, depending on the amount of polar lipids, and increase their size with increasing water content [18].

One key parameter for the uptake of color is porosity. In a hierarchical structure, porosity may occur at any size level. On the nanoscale, protofilaments form microtubules in the hair, which are hollow cylinders of about 25 nm diameter [5]. As they play a key role in intracellular transport [5], they might also be able to transport dye molecules into various sites within the nanostructure of hair. Between macrofibrils, fused borders were identified by changes in the filament arrangement pattern [16], which could be the origin of porosity in the 100 nm range, and on the micrometer scale, porosity may arise from the three basic structural features, the inner medulla, the cortex and the outer cuticle. As determined by microbeam SAXS in a synchrotron radiation source, these three regions not only differ in the orientation of the IF in the plane perpendicular to the hair axis—the cuticle is much higher oriented than the disordered cortex and medulla—but also, α-keratin seems to be present in the cuticle rather than β-keratin [19]. The transition from α-helices to β-sheets in keratin can be induced by mechanical loading. The keratin network has an important role in influencing and improving mechanical properties of hair [20]. This combines the macroscale (mechanical loading of the whole fiber in a tensile test) with the nano- and atomistic scale (the organization of the keratin network and the α-β-transition as a conformational change of coiled proteins).

The α-β-transition starts at roughly five per cent macroscopic strain and further transformation takes place until about 20 per cent macroscopic strain [21]. To follow the α-β-transition in-situ in water under strain, micro-Raman spectroscopy was used [9]. The effect of strain rate, humidity and temperature on the α-β-transition was investigated and a transformation ratio was found for the part of the transformed α-helices, as the Ifs consist of periodic α-helical and non-helical regions [22].

Structural features after mechanical loading are visible in the scanning electron microscopy (SEM): fracture surfaces show a completely different appearance, depending on the strain rate. Additionally, the surface morphology is strongly strain dependent and exhibits much stronger lifting of edges of the scales of the cuticle at higher strain [22]. The scales play an important role for the uptake of color by different types of hair: lifting of the scales of the cuticle like a pine cone is also used, to foster the penetration of dyes through the cuticle into the cortex. However, this procedure involves a chemical step, the use of ammonia. This might induce damage to the outer layer and usually results in a higher potential for adsorbing water [23]. It weakens hydrogen bonds, which makes the hair less flexible, more brittle and in general more likely to break [23].

A careful comparison of human and yak belly hair should take into account the hierarchical structure. Thus we decided to use Raman spectroscopy, SAXS, microscopy (SEM and OM), porosity measurements and tensile tests, as methods covering a broad range of properties from the nano- to the macroscale, i.e., from the molecular level to the size of the whole hair. The main goal is to analyze the similarities and differences of both types of hair, bearing in mind the suitability to replace human hair by yak belly hair in applications in cosmetics industries.

## 2. Results

### 2.1. Colorimetry

**Figure 2:**
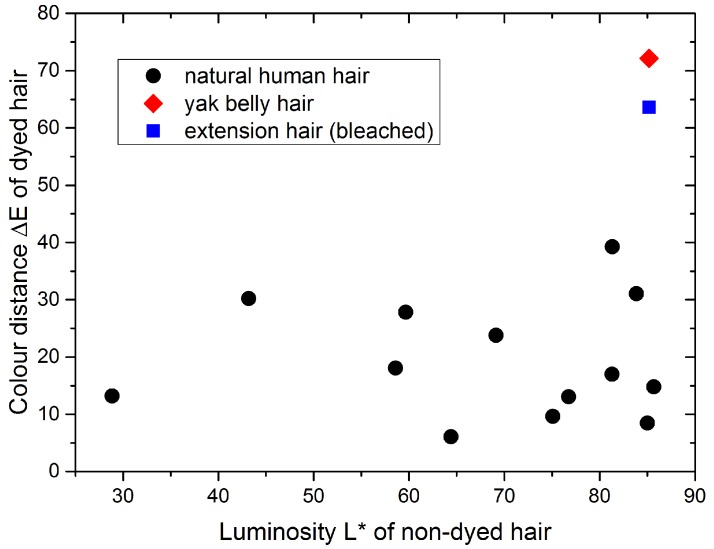
Color distance (after application of the same natural dye) of hair with different initial colors is rather constant for human hair (low value of luminosity: dark hair, high value of luminosity: bright hair). Extension hair, which is bleached (blue square), and yak belly hair, which lacks melanin (red diamond), exhibit a significantly higher color distance.

One of the most widely used ways to style one’s appearance is the coloring of hair with suited hair dyes. To assess the quality in different test procedures, it is therefore essential to quantify the color change, due to a specific treatment with a hair dye. We used a natural dye (logwood), and quantitatively measured the color distance for hair with different original colors. The color distance is calculated from UV-VIS spectroscopy in the CIELAB color space. This method was also used in previous works to determine the color change in human hair [24,25]. We emphasize that—for a comparison of human and yak belly hair—it is important to have a quantitative value for the uptake of the dye. As shown in Figure 2, the color distance (i.e., the uptake of the dye) is rather constant, and independent of the initial color of natural hair, which is shown on the abscissae. A lower value of the luminosity means darker and a higher value means brighter hair. Differently, extension hair, which is bleached, and yak belly hair, which lacks melanin, are on the one hand very bright and exhibit on the other hand a much stronger color distance, which is a clear sign of a higher uptake of the hair dye than is the case for natural human hair.

### 2.2. Raman Spectroscopy

**Figure 3:**
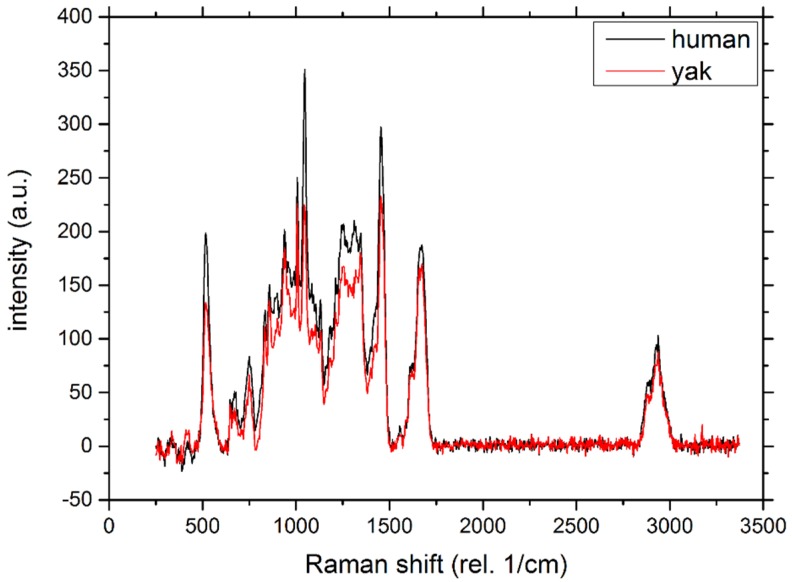
Raman spectra of human hair (black line) and yak belly hair (red line). There is a clear coincidence of all measured peaks of both spectra.

Typical Raman spectra of human (black line) and yak belly hair (red line) are shown in Figure 3. The spectra are nearly identical, and there is an obvious coincidence of all peaks. Differences in intensities arise from data normalization. The most prominent peaks are assigned to the S-S vibration at 509 cm^−1^, the S-O peak between 1020 and 1070 cm^−1^, the CH_2_ bending mode at 1450 cm^−1^, the Amide I band at about 1680 cm^−1^ and the CH_2_, CH_3_ stretching modes close to 3000 cm^−1^ [7,9,10,26]. Therefore, from a chemical point of view, human and yak belly hair are very similar. It was this distinct molecular similarity, which suggested that yak belly hair could perfectly substitute human hair [7].

### 2.3. Nanostructure from Small-Angle X-ray Scattering (SAXS)

**Figure 4:**
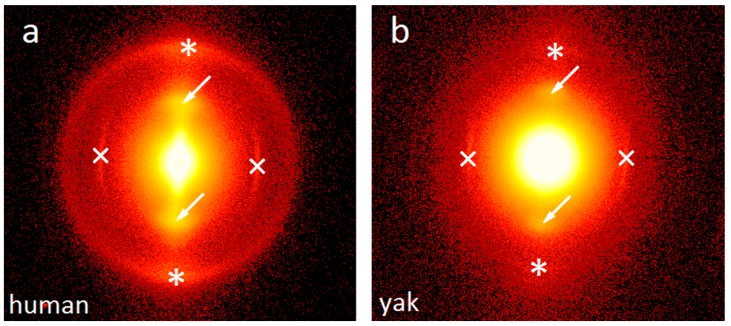
Two-dimensional small-angle X-ray scattering (SAXS) patterns of (**a**) human and (**b**) yak hair. The intermediate filament distance peak is indicated by arrows, the peak from the axial stagger of molecules by crosses, and the lipid bilayer ring by asterisks. The latter is very weak in yak belly hair. The much higher scattering intensity towards the center is induced by a much higher porosity in yak belly hair.

X-ray scattering is an important tool to characterize the nanostructure of hair, in particular to determine the distance of the IF [13,14,17,27], the axial stagger between molecules or group of molecules along the microfibril [11,28], the lipid lamellae distance [18] and the structure across the cross-section of hair by microdiffraction [19,29]. Figure 4 shows typical SAXS patterns from bundles of human and yak belly hair to visualize similarities and differences: for both types of hair, the intensity maximum caused by the typical distance of IF (indicated by arrows) is present, but slightly less pronounced for human hair. The lipid bilayer ring (indicated by an asterisk) is clearly visible in human hair, but rather weak in yak belly hair, and the intensity maximum from the axial stagger of molecules (indicated by a cross) is found in both types of hair. An important feature is the strong scattering intensity towards the center (low *q*-values) in yak belly hair, which is caused by a significantly higher porosity.

**Figure 5:**
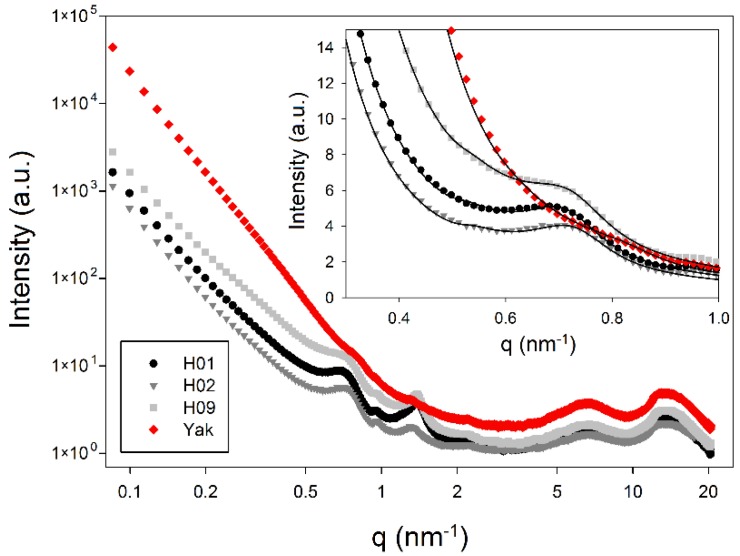
Integrated SAXS and wide-angle X-ray scattering (WAXS) intensities in dependence on the scattering vector *q*. The scattering intensity towards small *q*-values is significantly higher for strands of yak belly hair (red line) than for strands of human hair (black and grey lines, named by *H01*, *H02* and *H09*). The insert shows the data on a linear scale with fit curves from the paracrystalline model [27,32]. The model parameters (mean intermediate filament (IF) distance *<a>* and lattice parameter *d*, core radius *R*, and paracrystalline distortion Δ) together with axial molecule stagger and lipid layer distance are found in Table 1.

In Figure 5, SAXS and WAXS intensities are plotted in dependence on the scattering vector *q*. For the SAXS measurement, the same number of single hairs was used (20 single hairs) for each measurement, but, as yak belly hair is thicker than human hair, a volume normalization was performed (data were normalized by the transmission measured by a transparent beamstop). As in Figure 4, one observes that the IF distance peak is less pronounced, the axial stagger peak is similar, and the lipid bilayer ring is very weak in yak belly hair. Towards low *q*-values, the higher scattering intensity caused by the higher porosity of yak belly hair is clearly visible. Though this SAXS intensity masks the IF distance peak at about 0.7 nm^−1^, its peak position at a lower *q*-value, and its broader shape clearly suggest that the IF filaments are less ordered in yak belly hair. A complete disorder is excluded, as still a weak peak is visible. The high SAXS intensity in yak belly hair is roughly ten times higher than the one of human hair (data are plotted on a logarithmic scale). For an evaluation of typical IF distances and size of molecule core *R*, we used the paracrystalline model, which was originally developed for high polymers [30]. The paracrystalline concept was further developed to describe the organization of the IF filaments in keratin fibers [31,32] and the specific lateral assembly of the IF in two tetrameric oligomers [27]. The model delivers three parameters: the average microfibril diameter (the molecular core) *2R*; the mean filament distance in the hexagonal lattice *<a>,* which is related to the hexagonal lattice parameter (first peak position) *d* by *<a> = 2d/√3*; and the lattice distortion *∆* (standard deviation of *<a>*). For the calculation of the average interference function, different definitions for the basic lattice vectors were used [32,33,34], where we adopted the one presented in [34] for our calculations. In Figure 5, experimental SAXS and WAXS data are shown on a logarithmic scale as symbols (red: yak belly hair, black and grey: human hair), and in the insert of Figure 5, the predictions from the model are shown as lines (detailed image is plotted on a linear scale). Numerical results from the model fits are given in Table 1. The most striking difference in the SAXS regime is that yak hair has a significantly smaller IF distance (by about 10 per cent). As the microfibril diameter is of a comparable size in all hair types, this leads to the conclusion that the matrix thickness is considerably smaller in yak hair. A further difference, which is clearly visible in Figure 5, is the higher scattering intensity of yak belly hair towards small *q*-values, with a power-law background parameter of *n* = 4 in Table 1. It is very probably that high porosity with a pore size in the >100 nm regime induces this strong *q*^−4^ decrease of the scattering intensity. Other parameters, such as the values for the axial stagger distance are close to each other, and the lipid layer distance lies within the error margins. The error given is the standard deviation of the measurements for the human hair samples (*H01*, *H02*, and *H09*), and the fit parameter error for the yak sample, with each of the values obtained from a bundle of 20 strands. Both errors are of similar magnitude.

In the WAXS regime, scattering intensities of human and yak hair do not differ considerably, visible by the same shape and position of the peaks at high *q*-values in Figure 5. There is a slightly higher general scattering intensity in yak hair in the large *q*-range, maybe induced by a higher porosity in the subnanometer regime.

### 2.4. Mechanical Properties

Tensile tests were performed to compare mechanical properties of human and yak belly hair. The test procedure is described more precisely in the methods section: summarizing, a bundle of 30 single hairs was taken out of a strand of hair and prepared between two paper sheets, with a defined gauge length for strain evaluation. The hair bundle was clamped, and a force-displacement diagram was recorded. The strain was calculated from the crosshead displacement and the gauge length, the stress by dividing the force through the total area of the hair bundle. As described in the methods section, the latter was determined by optical microscopy (OM).

**Figure 6:**
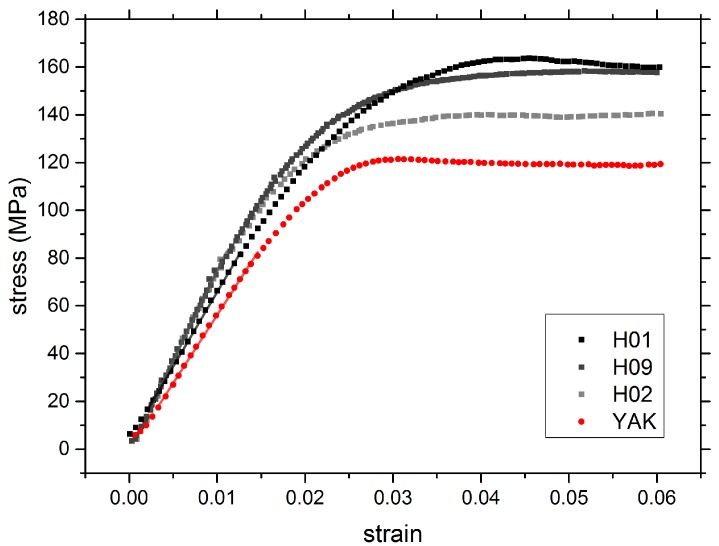
Stress-strain curves of human (black and grey squares) and yak belly hair (red dots). Both values, the Young’s modulus from the initial linear part of the stress-strain curve, as well as the maximum stress are lower for yak belly hair.

In Figure 6, the stress-strain curves of hair bundles from human (black and grey squares) and from yak belly hair (red dots) are depicted, and numerical values are given in Table 2. The most important features are the higher Young’s modulus of human hair (evaluated from the initial linear part of the stress-strain curve), and the higher maximum stress with increasing strain. There is a considerable variation between different human Caucasian hairs, but the lower value for the Young’s modulus of yak belly is obvious. Likewise, the maximum stress of yak belly hair is lower than the one of human hair. These are original data: if one corrects the Young’s modulus for density/porosity (taking numerical values from Section 2.5), the difference between yak belly hair and human hair nearly disappears.

### 2.5. Density

**Figure 7:**
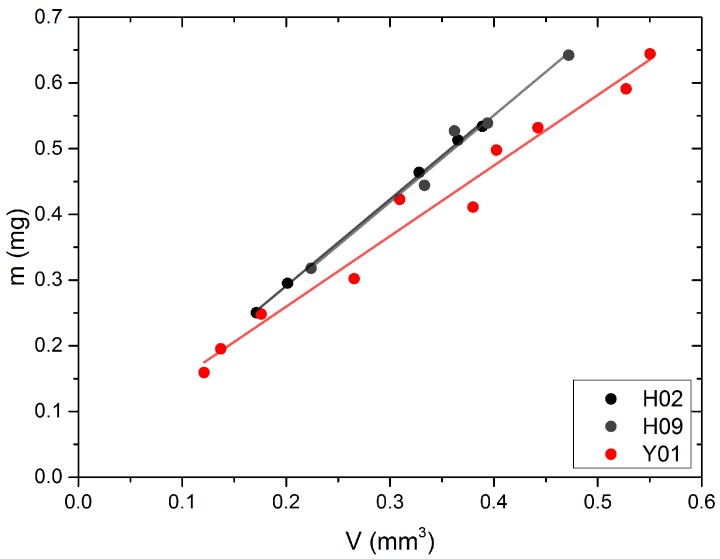
Weight vs. volume for human hair (black dots) and yak belly hair (red dots). The lines are fits and the density is obtained from the slope. This slope (i.e., the density) is lower for yak belly hair than for human hair.

SAXS data already evidenced that the nanoporosity of yak belly hair is significantly higher than that of human hair. Concerning macroporosity, density measurements were carried out. The density was calculated from the weight, determined by a microbalance, and the volume, where the cross-section was determined by rotating the samples in a laser scan micrometer (see methods section). Density of samples is then obtained from the slope in a weight vs. volume diagram (Figure 7). The slope (i.e., the density) is obviously lower for yak belly hair. Numerical values for the density are *ρ_yak_* = (1.074 ± 0.060) g/cm^3^ for yak belly hair and *ρ_human_* = (1.312 ± 0.043) g/cm^3^ for human hair.

### 2.6. Optical Microscopy (OM) and Scanning Electron Microscopy (SEM)

**Figure 8:**
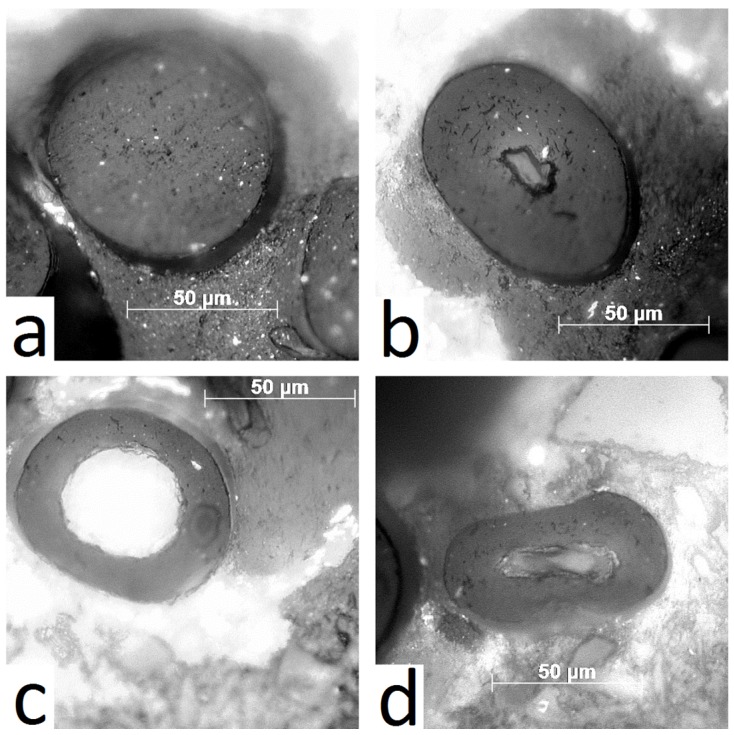
Four different appearances of cross sections of yak belly hair: (**a**) dense, (**b**) small tubular medulla, (**c**) large tubular medulla, (**d**) deformed large tubular medulla. In human hair, the majority is of type (**a**), dense, whereas in yak belly hair the majority of hairs has a large medulla, i.e., a tubular appearance with a large central channel (**c**).

One reason for the different porosity is visible in Figure 8a–d: yak belly hair frequently features large channels in the center, visible in cross-sections by OM. Only very rarely, dense hair (Figure 8a) is found, whereas most of the hairs include small holes and a large medulla (Figure 8b), or they appear even tube-like (Figure 8c) or tube-like and deformed (Figure 8d). From a total of 24 cross-sections, five were dense, six featured a small and 13 a large medulla. Differently, from a total set of 29 cross-sections of human hairs, 15 were dense, 11 had a small and only three a large medulla.

**Figure 9:**
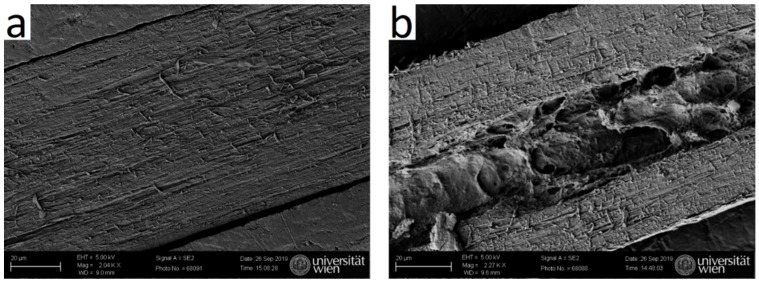
Scanning electron microscopy (SEM) image of a cut along the length axis of hair. Whereas (**a**) typical human hair is rather dense, (**b**) typical yak belly hair has a large central channel filled with foam-like material.

**Figure 10:**
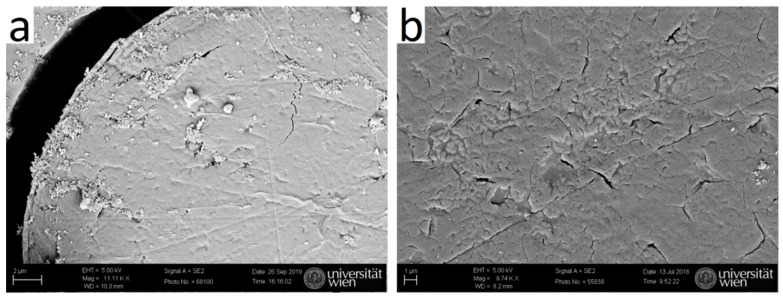
SEM image of polished cross-sections of (**a**) human and (**b**) yak belly hair. In yak belly hair, much tinier structures and cracks are visible than in human hair.

**Figure 11:**
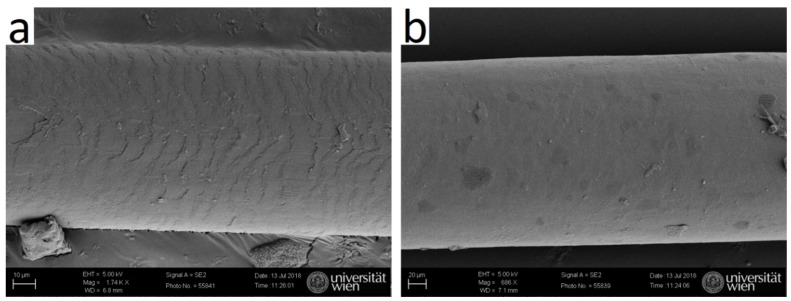
The surface of the cuticle appears scale-like in (**a**) human hair and smooth in (**b**) yak belly hair.

Cuts along the length axis were performed, and SEM micrographs were taken, to visualize the inner channel. SEM images show that typical human hair is rather dense (Figure 9a), whereas typical yak belly hair exhibits a large inner channel, which is filled with foam like tissue (Figure 9b). In polished cross-sections, the appearance of both hair types also differs considerably: whereas human hair appears rather smooth (Figure 10a), apart from some few cracks arising probably from drying after the polishing step, yak belly hair shows a multitude of cracks and pits (Figure 10b). Structures in yak belly hair are rather tiny, in the submicron range, and the crack opening is even much smaller, which makes these cracks probably the cause of the observed strong scattering in SAXS in Figure 4 and Figure 5. Figure 11 compares the surfaces of human and yak belly hair: in human hair (Figure 11a), the cuticle exhibits a large number of scales at the surface, whereas in yak belly hair, the surface appears to be smooth (Figure 11b).

## 3. Discussion and Conclusions

From the nearly identical Raman spectra, one can conclude that—from a molecular point of view—there is a clear coincidence of yak belly and human hair. The WAXS signal is also almost identical, which is consistent with the interpretation that the keratin fibrils are very similar. On the nanoscale, concluded from the SAXS measurements, most of the structural features are of similar magnitude, only the distance of the IF is considerably smaller in yak belly hair. As the core radius from the paracrystalline model is in the same size range for both types of hair, one can conclude that the difference arises from the considerably smaller thickness of the matrix (1.3 nm for yak belly hair in comparison to 2.9 nm for human hair, Table 1). The calculation of the matrix thickness is based on a hexagonal paracrystalline model, from which IF distance and the core radius can be obtained [32]. If the IF were organized not in a hexagonal lattice, but in another molecular architecture, as modelled for different assemblies of oligomers in Rafik et al. [27], one would obtain peaks in the scattering pattern at a different position. However, this cannot explain the smaller IF distance in yak belly hair, as, in general, a change from a two-dimensional hexagonal to other lattices would lead to higher IF distances. This would be the opposite as observed for yak belly hair. Therefore, the most probable interpretation of the data is that there is indeed a smaller matrix thickness in yak belly hair than in human hair, together with a lower order of the filaments. For the core radius, the value for human hair was *R* = 3.4 nm and *R* = 3.54 nm for yak belly hair. These values lie between their error margins, but they are about eight per cent smaller than the literature values [27]: a value for the diameter of 7.4 nm (radius 3.7 nm) was published for keratins of different species (human hair, horse hair, porcupine quill) in ambient conditions. In an early work by Fraser and McRae [35], a value of 3.65 nm was obtained using electron microscopy. Later, Parry [36] mentioned that a model with a core radius value of 3.47 nm and an outer radius of 4.39 nm would fit to the data, as well as the solid cylinder model, with a core radius of 3.65 nm. Briki et al. observed that the latter model is better suited to fit data from porcupine quill [27]. As the form factor of both models is rather similar, it is very difficult to distinguish them on the basis of the experimental SAXS data. A further reason for the slightly smaller values of the core radius in our evaluation could be the subtraction of the power-law background *q^-n^*: in our approach, the exponent *n* was a fit parameter, whereas in [27,37] it was determined by heating hairs fibers to a high temperature (235 °C), where the peaks disappeared. The remaining power-law scattering with *n* = 2.33 [27] was used as background. The reason why we have chosen *n* as free fit parameter was that we wanted an identical evaluation of all hair types, and yak belly hair had obviously—due to the high porosity—a much higher value for *n*. The fit gave a result close to 4, which indicates two distinct phases (i.e., porous material with a smooth surface). This procedure might be a reason for the in general smaller core radii in our work. However, despite the different background subtraction, the statement that IFs have a smaller distance, due to a smaller matrix thickness *m* in yak belly hair (*m* = 1.3 nm) in comparison to human hair (*m* = 2.9 nm) clearly holds. It coincides with differences between horsehair (*m* = 1.2 nm) and human hair (*m* = 2.2 nm) in [27].

One remarkable difference between horsehair in [27] and yak belly hair in our work is the strong *q^−4^* background in the latter. This is obviously due to a significantly higher porosity. As for yak belly hair, the scattering intensity decrease ranges widely into the SAXS regime, it is very probable that it is induced by pores of nanometer or submicron size. The typical pore size is in any case larger than about 50 nm, the limit of the accessible range of our data. However, the precise number of the nanopore size cannot be determined from our SAXS data. In the SEM images in Figure 10, a large number of tiny cracks are visible, which could also be the interface between macrofibrils in the submicron size. They are a possible origin of the strong SAXS intensity decrease in yak belly hair, as this structural feature is missing in human hair, which appears much denser. However, one should be cautious with this interpretation: though both types of hair were treated identically, a different sensitivity to drying processes during and after polishing cannot be excluded. Porosity of yak belly hair is also much more pronounced at the macroscale: this is not only visible in the existence of a medulla, which gives most of the samples from yak hair a tubular appearance, as shown in Figure 8 and Figure 9. Likewise, a lower density was also observed in general (Figure 7). One may speculate that medulla, as well as pores, lead to a decrease of the thermal conductivity, thus improving the isolation properties of hair. This would be advantageous for yaks, a species native to cold and harsh environment. Finally, the lower density induces a lower value of the Young’s modulus—but exceptional mechanical properties of hairs and furs would probably not be an evolutionary advantage. If one corrects the elastic modulus for porosity, the difference between human and yak hair vanishes nearly completely. This also supports the observations from Raman scattering that, in particular for the IF filaments, human and yak belly hair are very similar concerning their molecular and mechanical properties, but the porosity of yak belly hair is higher at all length scales. Though some of our results are only qualitative, quantitative numbers were given for a number of differences between the two hair types at different length scales, i.e., a higher color distance after application of dyes, the smaller IF distance and matrix thickness and the lower Young’s modulus and density of yak belly hair in comparison to human hair.

**Figure 12:**
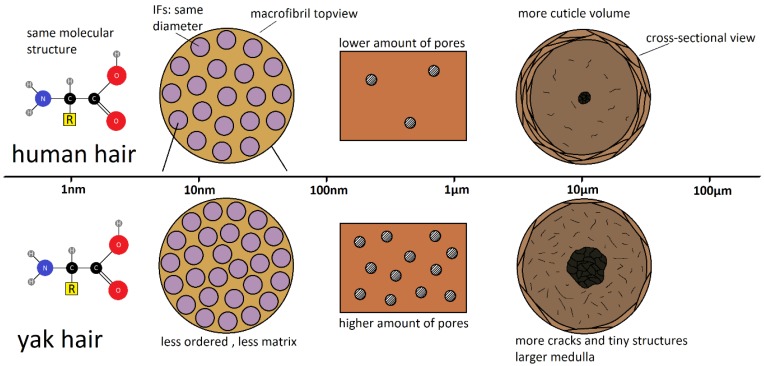
Scheme to visualize structural similarities and differences between human and yak belly hair.

In conclusion, three reasons lead to the observed higher color difference of yak belly hair in comparison to human hair, which are schematically shown in Figure 12: the main reason is porosity, which is more pronounced for yak belly hair from the nano- to the macroscale. The second reason is that yak belly hair is covered with a smaller amount of scales than human hair (visible in Figure 11) and the third is the lack of melanin. We tried to localize the uptake of the dye and correlate it to the microstructure, but its fluorescence was too strong, even at the largest available wavelength of the Raman microscope of 785 nm. Localization, as well as in situ following the uptake process, will be the theme of future research. Nevertheless, higher porosity and the absence of scales at surface lead to a much stronger uptake of hair dye, and increase the effectivity of the coloring process. On the one hand, this can be seen as an advantage, as a smaller amount of hair dye already has a strong effect. On the other hand, it can also be seen as a disadvantage, as the different effectivity in the coloring process limits the suitability to replace human with yak belly hair.

## 4. Materials and Methods

### 4.1. Preparation

Natural hair and yak belly hair samples were bought from commercial suppliers (yak hair: L’IMAGE GmbH; Natural hair: ADI Beautyextensions; Extensions: Real Russian Hair). All types of hair were cleaned by twice, washing with commercial shampoo and rinsing with running water. The hair strands were then divided into smaller bundles for different treatments. One untreated sample was always left as control. All samples were uniquely labelled with a code to identify the respective hair sample, treatment dye, time, temperature, and test conditions.

### 4.2. Colorimetry

From each sample, bundles of approximately 20 hairs were scanned in a Cary 5G UV-VIS spectrophotometer (Agilent, Santa Clara, CA, USA) in a wavelength range of 300 nm to 900 nm in transmission mode. As indicated by the intensities in dependence on wavelength, the spectra were normalized under the assumption that hair completely absorbs light at low wavelengths, whereas it is completely transparent for high wavelengths. This made it possible to calculate the color coordinates in the CIELAB color space L*, a* and b*, which consists of values for the luminosity L* (ranging from black to white) and parameters a* (axis ranging from green to red) and b* (axis ranging from yellow to blue). The color distance by any hair treatment is then defined as the length of a vector between untreated and treated points in CIE-Lab space [24,25]:

(1) $$\Delta E=\sqrt{{\left(\Delta L*\right)}^{2}+{\left(\Delta a*\right)}^{2}+{\left(\Delta b*\right)}^{2}}$$

The symbol *∝* in Equation (1) characterizes the difference of the respective parameter to the one of untreated hair.

### 4.3. Raman Spectroscopy

Measurements were performed with a WITEC alpha 300RA combined confocal Raman and AFM instrument (WITEC, Ulm, Germany). Single hairs were fixed on a glass sample holder with scotch tape, and measured for 200 s with a laser wavelength of 785 nm at a power of 60 mW. Measurements were either performed in depth direction by moving the focal point vertically through the sample, or in lateral direction with line scans at surfaces of polished cross-sections. The first method has the advantage that no preparation step has to be performed, which could eventually have an effect on the Raman signal, whereas the second method has the advantage of a higher positional resolution.

### 4.4. SAXS and WAXS

SAXS and WAXS measurements were performed with Cu-Kα radiation (*λ* = 0.1542 nm) from a Bruker Nanostar (Bruker AXS, Karlsruhe, Germany), equipped with a pinhole camera and a 2D detector (VÅNTEC 2000) at two different sample to detector distances (108 cm and 11 cm). The data were linked together in the overlapping region. All data were background corrected, azimuthally integrated and normalized to the transparent beamstop to obtain the scattering intensity in dependence on the scattering vector *q*, being defined as *q =* (*4π/λ*)*·*sin*ϑ*, with *2ϑ* being the scattering angle. The accessible *q*-range was between 0.1 and 20 nm^−1^. The measurement time for a hair bundle consisting of about 20 hairs (taken after the colorimetry measurements) was typically half an hour. To take a hair bundle for the measurements has two advantages, one is the shorter measurement time, and the other is that a single measurement gives a statistical average over different samples. In the SAXS patterns, three peaks were identified. The IF peak at about 0.7 nm^−1^ was fitted with the paracrystalline model [27,31,32], the axial molecule stagger peak close to 1 nm^−1^, and the lipid layer peak at about 1.3 nm^−1^ with Lorentzian functions.

### 4.5. Tensile Tests

Tensile tests were performed in a uniaxial tensile test device. The strain was measured from the crosshead displacement via a LVDT. The compliance of the load train was determined, but the compliance correction from the load train is negligible in comparison to the compliance of hairs. Thirty hairs were taken for one measurement to obtain a statistical average. This hair bundle was fixed to a paper frame and then glued for a length of at least 20 mm to avoid slipping. The gauge length was 10 mm and tests were performed with a crosshead speed of 0.01 mm per second.

The stress was calculated from the ratio of the force to the loaded area, which was determined by OM from the unstrained part of the bundle (the one glued between the papers). Cross sections were prepared by cutting and polishing. The individual area of each single hair was obtained by a polygon area, a built-in function of the Zeiss image evaluation software. The area from the single hairs was added to result into the total cross-sectional area of the hair bundle.

### 4.6. Density

Geometrical density was obtained from the weight and volume of individual hairs. Ten yak hairs and 10 human hairs (two times five hairs from different persons) were taken, and the weight was measured by a precision balance (Sartorius M3P, resolution 1 μg). The volume was calculated from the length and the cross-section. The length of each hair varied between 80 and 100 mm and was measured by a caliper. The cross section was determined each 5 mm along the length, by rotating the sample in steps of 10°, and measuring the diameter at each step with a laser scan micrometer (LSM 500S, precision 0.03 μm, Mitutoyo Corporation, Kawasaki, Japan). The total cross-section was then calculated from fitting ellipses to each single cross-section and calculating an average of this set of cross-sections.

### 4.7. SEM

Samples were coated with palladium-gold, and images were taken in a Zeiss Supra 55 VP scanning electron microscope in vacuum.

## Figures and Tables

**Table 1:** Structural features of human hair (average of *H01*, *H02*, *H09*) and yak hair on the nanoscale, determined by SAXS. The paracrystalline distortion Δ was close to 0.25 in all fits and thus set to this value as in [32]. The fit parameters were the lattice parameter *d* (mean IF distance *<a> = 2d/√3*), the core radius *R* and the power law background *I∝ q^-n^*. The matrix thickness is obtained by *m = <a > − 2R* and the axial stagger and lipid layer distance were calculated from fits with Lorentzian functions.

Measured Values	Human Hair	Yak Hair
Lattice parameter *d* (nm)	8.37 ± 0.10	7.27 ± 0.08
IF distance *<a>* (nm)	9.66 ± 0.11	8.40 ± 0.10
Core radius *R* (nm)	3.40 ± 0.09	3.54 ± 0.12
Matrix thickness *m* (nm)	2.86 ± 0.15	1.32 ± 0.15
Axial stagger distance (nm)	6.54 ± 0.05	6.65 ± 0.05
Lipid layer distance (nm)	4.62 ± 0.14	4.74 ± 0.10
Power law background *n*	2.9 ± 0.1	4

**Table 2:** Young’s modulus of human and yak hair.

Measured Value	Human (GPa)	Yak (GPa)
Young’s modulus	7.11 ± 0.44	5.76 ± 0.04
